# Iron chelation suppresses secondary bleeding after intracerebral hemorrhage in angiotensin II‐infused mice

**DOI:** 10.1111/cns.13706

**Published:** 2021-08-04

**Authors:** Jie Wang, Xiao‐qin Tang, Min Xia, Cheng‐cheng Li, Chao Guo, Hong‐fei Ge, Yi Yin, Bo Wang, Wei‐xiang Chen, Hua Feng

**Affiliations:** ^1^ Department of Neurosurgery Southwest Hospital Third Military Medical University (Army Medical University Chongqing China; ^2^ State Key Laboratory of Trauma, Burn and Combined Injury Third Military Medical University Chongqing China; ^3^ Chongqing Key Laboratory of Precision Neuromedicine and Neuroregenaration Southwest Hospital Third Military Medical University Chongqing China

**Keywords:** angiotensin II, cerebral vascular injury, intracerebral hemorrhage, iron overload, secondary bleeding

## Abstract

**Aims:**

Secondary bleeding and further hematoma expansion (HE) aggravate brain injury after intracerebral hemorrhage (ICH). The majority of HE results from hypertensive ICH. Previous study reported higher iron content in the brains of hypertensive patients. Iron overload exacerbates the risk of hemorrhagic transformation in thromboembolic stroke mice. Whether iron overload during the process of hypertension participates in secondary bleeding of hypertensive ICH remains unclear.

**Methods:**

Hypertension was induced by continuous infusion of angiotensin II (Ang II) with an osmotic pump into C57BL/6 mice. ICH was simulated by intrastriatal injection of the liquid polymer Onyx‐18. Iron chelation and iron overload was achieved by deferoxamine mesylate or iron dextran injection. Secondary bleeding was quantified by measuring the hemoglobin content in the ipsilateral brain hemisphere.

**Results:**

Ang II‐induced hypertensive mice showed increased iron accumulation in the brain and expanded secondary hemorrhage after ICH modeling. Moreover, iron chelation suppressed while iron overload aggravated secondary bleeding. Mechanistically, iron exacerbated the loss of contractile cerebral vascular smooth muscle cells (VSMCs), aggravated blood–brain barrier (BBB) leakage in Ang II‐induced hypertensive mice, and increased glial and MMP9 accumulation after ICH.

**Conclusion:**

Iron overload plays a key role in secondary bleeding after ICH in Ang II‐induced hypertensive mice. Iron chelation during the process of Ang II‐induced hypertension suppresses secondary bleeding after ICH.

## Graphical abstract

Iron accumulation in the brain tissues of Ang II‐induced hypertensive mice might exacerbate the loss of contractile VSMCs, enhance perivascular inflammation and BBB leakage. These factors might lead to increased vascular fragility and increased vulnerability to rupture, ultimately causing more secondary bleeding and subsequent HE after ICH.

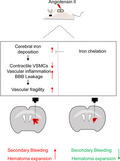

## 1 INTRODUCTION

The mechanisms contributing to acute hematoma growth in intracerebral hemorrhage (ICH) are not well understood. In 1971, Fisher introduced the theory of secondary bleeding and hematoma expansion (HE).^1^ Human data including the presence of spot sign (contrast extravasation within an ICH) on computed tomography angiogram,^2^ simultaneous extravasation from multiple vessels during cerebral angiography after ICH,^3^, ^4^ and capture of a developing hematoma by a magnetic resonance imaging scanner,^5^ support this theory. In a recent study, initial ICH was simulated by intrastriatal injection of a liquid polymer (Onyx‐18) that coagulates upon contact with tissue, and multiple foci of secondary hemorrhage mainly around the perimeter of the polymer, was observed, providing evidence of secondary vessel rupture.^6^

Iron is essential for cellular homeostasis because of its ability to donate and receive electrons.^7^, ^8^ However, under conditions of primary (hereditary) and secondary (acquired) iron overload, iron deposition might increase in different tissues.^9^ In the cardiovascular system, iron overload has been reported to augment angiotensin II (Ang II)‐induced cardiac fibrosis,^10^ aggravate atherosclerosis,^11^, ^12^ induce vascular dysfunction in resistant pulmonary arteries associated with right ventricular remodeling,^13^ accelerate thrombus formation after arterial injury,^14^ increase vascular oxidative stress, and impair vasoreactivity.^15^ In addition, higher iron status is associated with increased stroke risk.^16^ Iron overload exacerbates the risk of hemorrhagic transformation after tissue‐type plasminogen activator administration in thromboembolic stroke mice.^17^ Amelioration of post‐ICH iron overload caused by the hemoglobin lysis with iron chelators attenuates brain injury after ICH.^18^, ^19^, ^20^, ^21^ However, whether iron overload status before ICH contributes to secondary bleeding in hypertensive ICH remains unclear.

It has been reported that long‐term administration of Ang II in rats might cause accumulation of iron in the kidney,^22^, ^23^ heart,^10^ liver,^24^ and aorta.^25^ Thus far, little is known about whether Ang II induces abnormal iron homeostasis in brain tissues. Increased serum ferritin levels are common in men with essential hypertension.^26^ Significantly higher iron content in hypertensive patients than in normal subjects has been observed in all examined brain regions.^27^ Cortical superficial siderosis was shown to be an independent variable associated with a larger ICH volume in the lobar ICH.^28^ Therefore, we hypothesized that abnormal iron deposition might occur in the brains of Ang II‐induced hypertensive mice, and that iron accumulation might play an important role in cerebral vascular injury and hematoma enlargement after ICH modeling.

In the present study, by using a modified ICH mouse model, we investigated the effect of iron chelation and iron overload on secondary bleeding in Ang II‐induced hypertensive mice after ICH modeling and the possible mechanisms.

## 2 METHODS

### 2.1 Animals and experimental design

Healthy male C57BL/6J mice were purchased from the Experimental Animal Center at the Third Military Medical University (permit no. Yu2017‐0002; Chongqing, China) and used in the present study. All experiments complied with the ARRIVE guidelines^29^ and were carried out in accordance with the National Institutes of Health Guide for the Care and Use of Laboratory Animals (NIH Publications no. 8023, revised 2011).

All experimental animal procedures were conducted in accordance with the Institutional Animal Care and Use Committee at the Third Military Medical University (Army Medical University). All of our study protocols were approved by the Ethics Committee of the Third Military Medical University. This article does not contain any studies with human participants performed by any of the authors.

**FIGURE 1:**
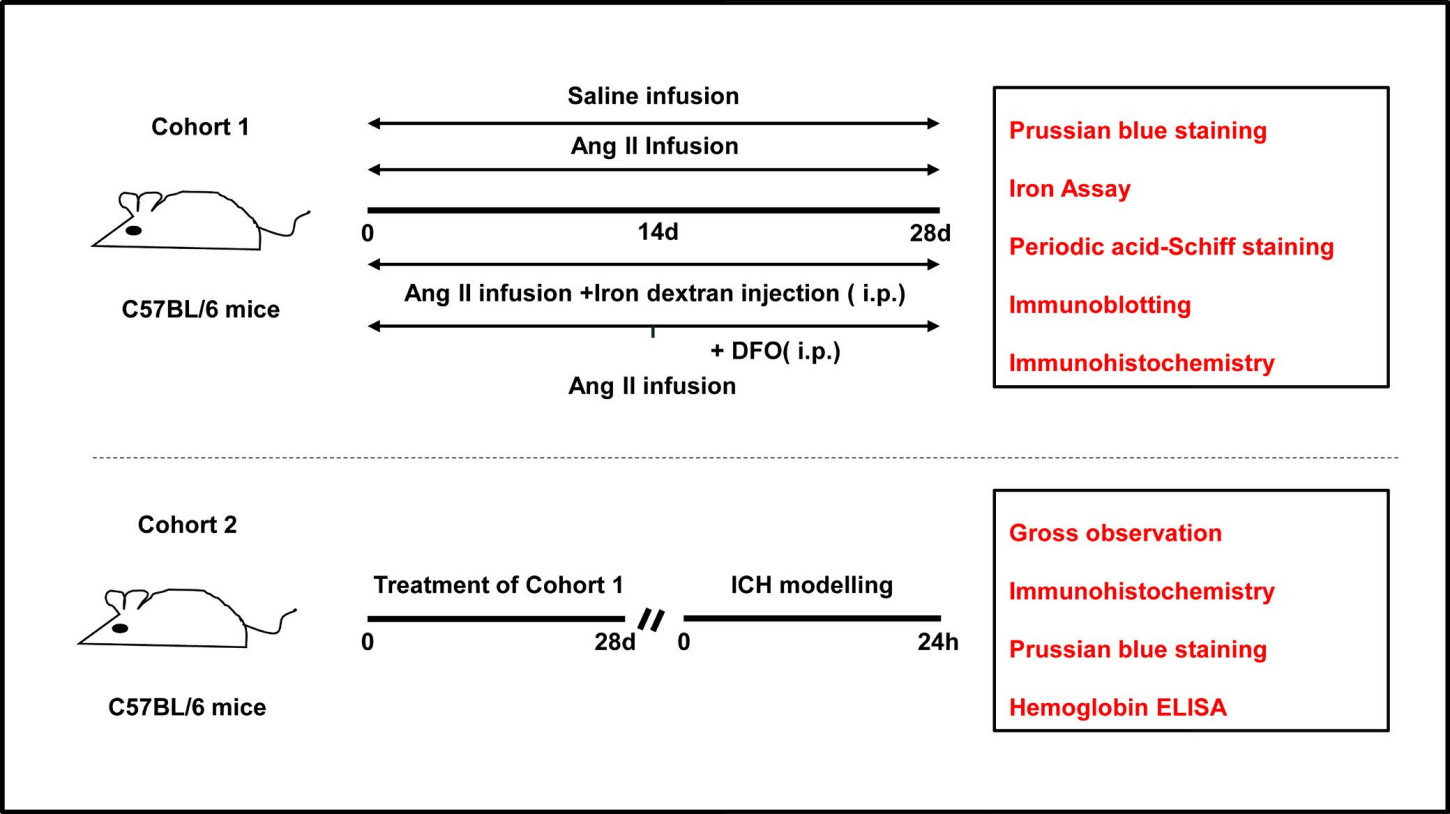
Flow chart of experimental design. Ang II, angiotensin II; DFO, deferoxamine mesylate; i.p., intraperitoneal injection; ICH, intracerebral hemorrhage

There were two cohorts of mice used in this study (Figure 1). The first cohort was used to determine whether iron accumulates in the brain and evaluate its effect on cerebral vascular damage in Ang II‐induced hypertensive mice. Mice in this cohort were treated with saline, Ang II, Ang II plus DFO (deferoxamine mesylate), or Ang II plus iron dextran. After treatment, tissue samples were collected for Prussian blue staining, iron assay, immunoblotting, immunohistochemistry, and periodic acid Schiff (PAS) staining. The second cohort was used to evaluate the role of iron in secondary bleeding in Ang II‐induced hypertensive mice after modified ICH modeling. After the mice received the same treatments as those in cohort 1, they were subjected to intrastriatal injection of the liquid polymer Onyx‐18 to simulate ICH. Twenty‐four hours after ICH modeling, brain samples were collected for gross observation, Prussian blue staining, immunohistochemistry, and hemoglobin enzyme‐linked immunosorbent assay (ELISA).

### 2.2 Animal model of angiotensin II‐induced hypertension

Adult male C57BL/6 mice were infused with Ang II (1,000 ng‐1 kg‐1 min‐1; Sigma‐Aldrich) using an osmotic pump (Durect Corporation) for 28 days as previously reported.^30^

### 2.3 Iron chelation and overload

The iron chelator deferoxamine mesylate (Sigma‐Aldrich) was intraperitoneally administered at a dose of 400 mg/kg per day for 14 days (during days 15–28 of Ang II administration). The iron overload model was established as previously described,^25^ briefly, mice received daily intraperitoneal injections of iron‐dextran (Sigma‐Aldrich) at a dose of 10 mg/mouse 5 days a week for 4 weeks beginning the same day as Ang II administration.

### 2.4 Animal model of modified intracerebral hemorrhage

A modified ICH mouse model was constructed by intrastriatal injection of the space‐occupying, nonblood liquid polymer Onyx‐18. Onyx‐18 gradually solidifies after contacting tissue to exert an ICH‐like mass effect and can be easily distinguished from secondary bleeding as reported previously.^6^ The animals were anesthetized with halothane (70% N_2_O and 30% O_2_; 4% for induction, 2% for maintenance, China) and immobilized on a stereotactic instrument (RWD Life Sciences Ltd.). The following coordinates were used for intrastriatal injection: from bregma: 0.8 mm anteriorly, 2.5 mm laterally, and 3.0 mm deep. After flushing with dimethyl sulfoxide, a 28‐gauge syringe was loaded with liquid polymer. The needle was slowly inserted into the striatum at a depth of 3.0 mm, and 2.5 μl of the polymer was injected at a rate of 0.5 μl/min with an infusion pump. After injection, the needle was removed. The craniotomy was sealed with bone wax, and sutures were applied to the scalp. During the entire experiment and recovery, the body temperature of each animal was maintained at 37 ± 0:5°C. Twenty‐four hours after ICH modeling, the mice were sacrificed, and brain samples were collected for further study.

### 2.5 Immunohistochemistry

The brains were removed after perfusion with the fixative 4% paraformaldehyde and then immersed in 30% sucrose in phosphate‐buffered saline (PBS). Serial sections were cut on a freezing microtome. The tissue sections were then incubated with 3% H_2_O_2_ for 10 min in the dark at room temperature to quench endogenous peroxidase activity. Antigen retrieval was then performed. Subsequently, the sections were blocked for 20 min at 37°C with normal serum from the same species as that from which the secondary antibody was derived. The sections were then incubated with primary mouse anti‐ferritin (diluted 1:500, Abcam, ab75973), anti‐Mac2 (diluted 1:500, Abcam, ab2785), rabbit anti‐Mcp‐1 (diluted 1:500, Abcam, ab7202), and mouse anti‐fibrinogen (diluted 1:200, Abcam, ab58207) antibodies at 4°C overnight. After washing with PBS for 15 min at room temperature, the sections were incubated with a horseradish peroxidase (HRP)‐linked secondary antibody at 37°C for 40 min, reacted with a 3,3′‐diaminobenzidine substrate solution (Dako Cytomation GmbH) and counterstained with or without Mayer's hematoxylin.

### 2.6 Immunofluorescence staining

The brains were removed after perfusion with the fixative 4% paraformaldehyde and then immersed in 30% sucrose in PBS. Serial sections were cut on a freezing microtome, blocked, and incubated with the following primary antibodies: goat anti‐CD31 (diluted 1:250, R&D, AF3628), rabbit anti‐Iba1 (diluted 1:500, Wako, 019‐19741), rat anti‐CD68 (diluted 1:100, Abcam, ab53444), rat anti‐C3 (diluted 1:100, Abcam, ab11862), mouse anti‐AQP4 (diluted 1:100, Abcam, ab9512), rabbit anti‐GFAP (diluted 1:500, Abcam, ab7260), and rabbit anti‐MMP9 (diluted 1:500, Abcam, ab38898). After washing, the sections were incubated with the appropriate Alexa Fluor 488‐ or Alexa Fluor 555‐conjugated secondary antibody (diluted 1:1000, Invitrogen) and counterstained with DAPI. Images were captured with a Zeiss microscope (Zeiss, LSM780).

### 2.7 Immunoblotting

Brain samples were collected and proteins were obtained with lysis buffer (Thermo Scientific) supplemented with protease inhibitors (Sigma‐Aldrich). After electrophoresis, the proteins were transferred to PVDF (polyvinylidene fluoride) membranes. The membranes were blocked by incubation with 5% dry milk for 2 h at room temperature and then incubated with primary antibodies against ferritin (diluted 1:1000, Abcam, ab75973), alpha smooth muscle actin (α‐sma, diluted 1:1000, Abcam, ab5694), TAGLN / transgelin (Sm22α, diluted 1:1000, Abcam, ab89989), and GAPDH (diluted 1:2000, zsbio, TA‐08) at 4°C overnight. After thorough washing, the membranes were incubated with HRP‐conjugated IgG (diluted 1:2000, Proteintech) for 2 h at room temperature. The protein bands were visualized using ECL kits (Thermo Scientific) and imaged with a ChemDoc MP imaging system (Bio‐Rad). Images of the blots were analyzed using ImageJ software (NIH).

### 2.8 Periodic Acid‐schiff staining

The brains were removed after perfusion with fixative 4% paraformaldehyde and then immersed in 30% sucrose in PBS. Serial sections were cut on a freezing microtome. Extravasated plasma in the brain was evaluated by PAS (Solarbio science & Technology) staining. Brain sections were washed three times with distilled water, incubated for 5–8 min` with an oxidizing agent, washed two more times with distilled water, and then incubated for 10–20 min with Schiff reagent. After washing, the sections were counterstained with hematoxylin for 2 min and acidic differentiation solution for 2–5 s. Positive PAS staining was purplish‐red.

### 2.9 Prussian blue staining

The brains were removed after perfusion with the fixative 4% paraformaldehyde and then immersed in 30% sucrose in PBS. Serial sections were cut on a freezing microtome. Iron oxide distribution was assessed with a Prussian blue staining kit (Solarbio Science & Technology). Brain sections were washed three times with PBS, incubated for 25–30 min with 10% potassium ferrocyanide, washed two more times with PBS, and counterstained with nuclear fast red for 10 min. Cells containing intracytoplasmic blue granules were considered Prussian blue positive (PB‐positive).

### 2.10 Quantification of hemorrhages

Twenty‐four hours after ICH modeling, the mice were transcardially perfused with PBS to clear intravascular blood. The brains were removed and cut into 1 mm coronal slices with a mouse brain matrix, and the solidified polymer was removed from the brain tissues. The tissue from the area between 3 mm anterior and 3 mm posterior to the needle track was divided into the left and right hemispheres, homogenized in cell lysate buffer, and centrifuged at 18,880 *g* for 20 min. Then, the ipsilateral hemispheres were subjected to analysis with a Mouse Hemoglobin ELISA Kit (Abcam).

### 2.11 Iron assay

The level of iron in the brain tissues was determined using an iron assay kit (Abcam, ab83366) according to the manufacturer's instructions. Twenty‐eight days after Ang II infusion, the mice were transcardially perfused with PBS under deep (5%) isoflurane anesthesia to clear the intravascular blood. The brains were excised, washed in cold PBS and homogenized in 5× volumes of iron assay buffer using a Dounce homogenizer on ice. After centrifugation (4°C, 16,000 *g*, 10 min), the supernatant was collected for the iron assay. The volume of 25 µl of each sample was made up to 100 µl with assay buffer in a 96‐well plate, and the samples and standards were incubated for 30 min at 37°C with 5 µl iron reducer. Then 100 µl of iron probe was added to each reaction, mixed, and incubated for additional 1 h at 37°C in the dark. The output was measured immediately on a colorimetric microplate reader (OD at 593 nm). Iron concentrations were calculated from the standard curve and normalized to the amount of protein in each sample, as determined by the Bradford protein assay. The average of technical triplicates was calculated for each biological sample.

### 2.12 Gross observation of secondary bleeding

Twenty‐four hours after ICH modeling, the mice were transcardially perfused with PBS followed by the fixative 4% paraformaldehyde. The brains were removed and then immersed in 30% sucrose in PBS. Serial sections were cut on a freezing microtome. Images of gross sections were captured with a digital camera (Nikon D7100), and representative images of secondary bleeding are included in the article.

### 2.13 Statistical analysis

All statistical analyses were performed using GraphPad Prism software. Quantitative data are expressed as the mean ± SEM. Shapiro Wilk normality test was used to assess distribution of the data. If the data were normally distributed, comparisons between two groups were carried out using two‐tailed Student's *t*‐tests. Comparisons between multiple groups were analyzed by one‐way ANOVA followed by the Scheffé *F* test for post hoc analysis or by Student's *t*‐test. If the data were not normally distributed, analysis was performed using nonparametric statistics. Differences were considered statistically significant at *p* < 0.05.

## 3 RESULTS

### 3.1 Ang II infusion increased iron accumulation in the brain

**FIGURE 2:**
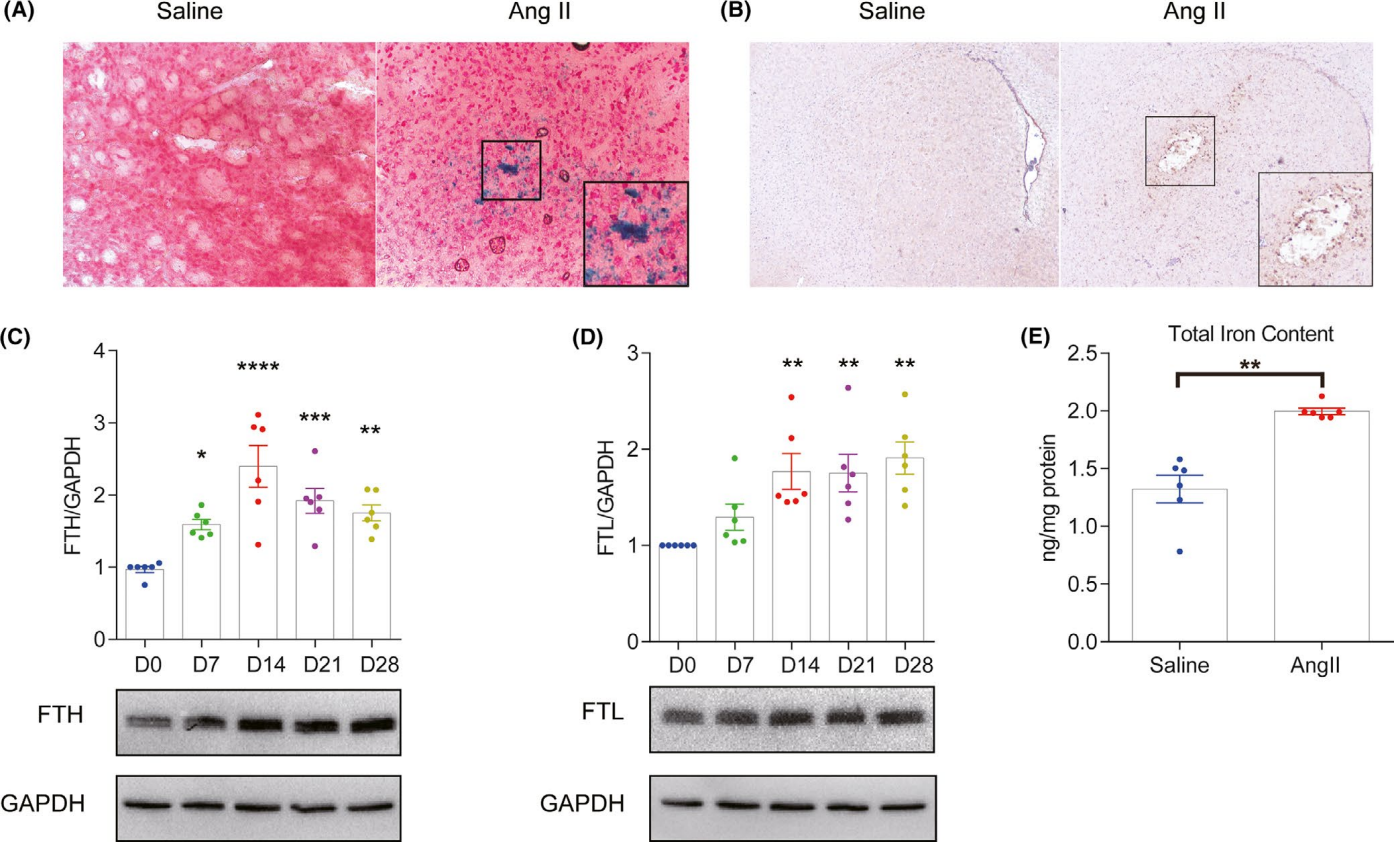
Angiotensin II (Ang II) infusion enhanced iron deposition in brain tissues. (A) Representative images of Prussian blue staining at 28 days after Ang II/saline infusion. (B) Representative images of ferritin immunochemistry staining at 28 days after Ang II/saline infusion. (C/D) Western blot analysis and quantification of the ferritin heavy chain and ferritin light chain protein levels in the brains of Ang II‐infused mice at 0, 7, 14, 21, and 28 days after Ang II infusion (*n* = 6). (E) Total iron levels in the mouse brain at 28 days after Ang II/saline infusion (*n* = 6). *p < 0.05, **p < 0.01, ***p < 0.005, ****p < 0.001

Increased iron accumulation was observed in the brain tissues of Ang II‐induced hypertensive mice. Prussian blue staining and ferritin immunochemistry showed that iron deposits were scattered in the brain parenchyma and the vascular wall (Figure 2A,B). The protein levels of ferritin heavy chain and ferritin light chain also increased significantly during Ang II infusion (Figure 2C,D). In addition, total iron levels in the brain increased from 1.32 ng/mg to 1.99 ng/mg of protein (Figure 2E).

### 3.2 Iron chelation during the process of Ang II‐induced hypertension attenuated secondary bleeding after ICH

**FIGURE 3:**
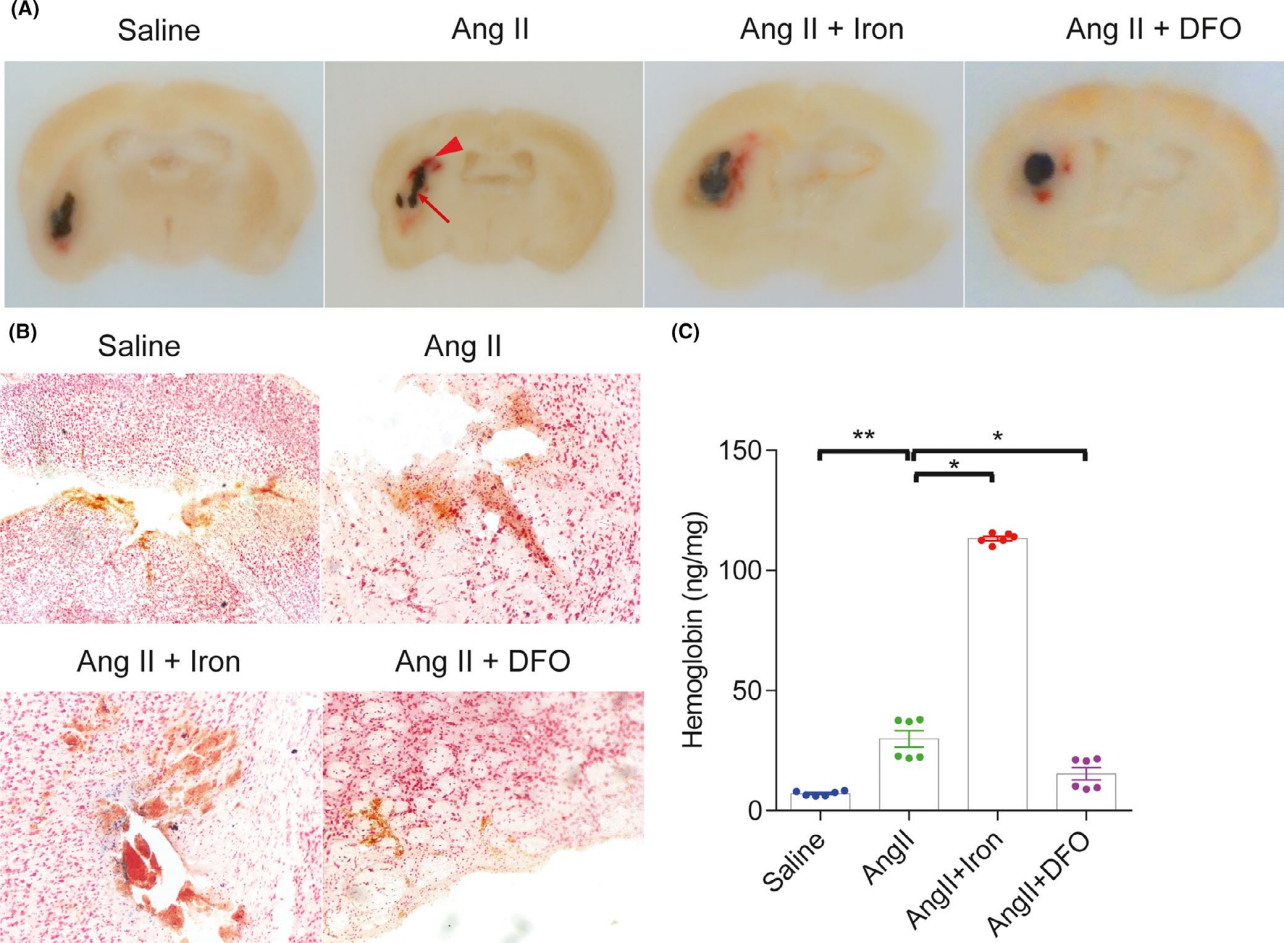
Iron chelation attenuated secondary hemorrhage in Angiotensin II‐induced hypertensive mice after intracerebral hemorrhage (ICH). (A) Representative images showing the solidified polymer (arrow) and secondary bleeding (arrow head) at 24 h after ICH modeling. (B) Representative images of Prussian blue staining showing the iron deposition (blue) at the bleeding sites at 24 h after ICH modeling. (C) At 24 h after ICH, hemoglobin levels in the ipsilateral hemisphere encompassing the injection site were measured by enzyme‐linked immunosorbent assay. *n* = 6 per group. *p < 0.05, **p < 0.01, ***p < 0.005, ****p < 0.001

After ICH modeling, more secondary bleeding (arrow head) was observed around the black solidified polymer (arrow) in the Ang II‐infused mice than in the saline‐infused mice. To investigate whether iron accumulation was involved in the secondary bleeding in the Ang II‐induced hypertensive mice after ICH, iron chelation/overload was induced. Iron overload further increased secondary bleeding. In contrast, iron chelation significantly decreased secondary bleeding (Figure 3A). Prussian blue staining demonstrated that iron deposition was increased around the area of secondary bleeding (Figure 3B). Quantification of secondary bleeding by hemoglobin ELISA further validated the effect of iron intervention on secondary bleeding (Figure 3C).

### 3.3 Iron chelation during the process of ang II‐induced hypertension attenuated loss of the cerebral vascular smooth muscle cell contractile phenotype

**FIGURE 4:**
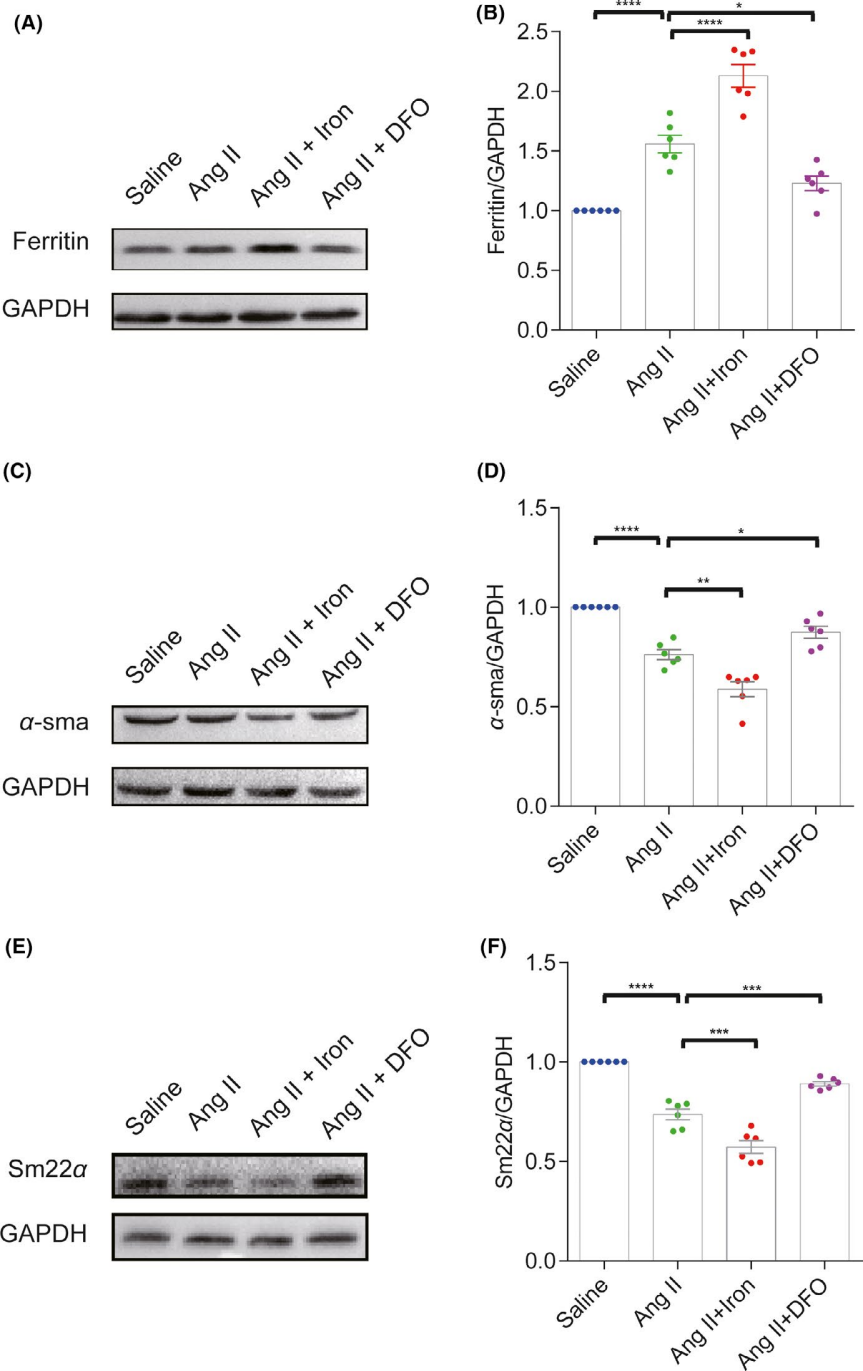
Iron chelation rescued the loss of the cerebral vascular smooth muscle cell contractile phenotype in angiotensin II (Ang II)‐induced hypertensive mice. Western blot analysis and quantification of ferritin (A and B), α‐sma (C and D) and Sm22α (E and F) protein levels in mouse brain at 28 days after saline, Ang II, Ang II +iron dextran or Ang II +DFO treatment (*n* = 6). *p < 0.05, **p < 0.01, ***p < 0.005, ****p < 0.001

To explore the possible mechanisms by which iron deposition enhanced secondary bleeding, the influence of iron on vascular smooth muscle cells (VSMCs), key contributor to the vascular integrity, was investigated. The levels of the canonical VSMC contractile markers α‐sma and Sm22α, decreased significantly in Ang II‐induced hypertensive mice. Iron overload further enhanced the downregulation of the expression of these two markers, while iron chelation rescued the loss of α‐sma and Sm22α (Figure 4).

### 3.4 Iron chelation during the process of Ang II‐induced hypertension attenuated leukocyte recruitment and blood–brain barrier leakage

**FIGURE 5:**
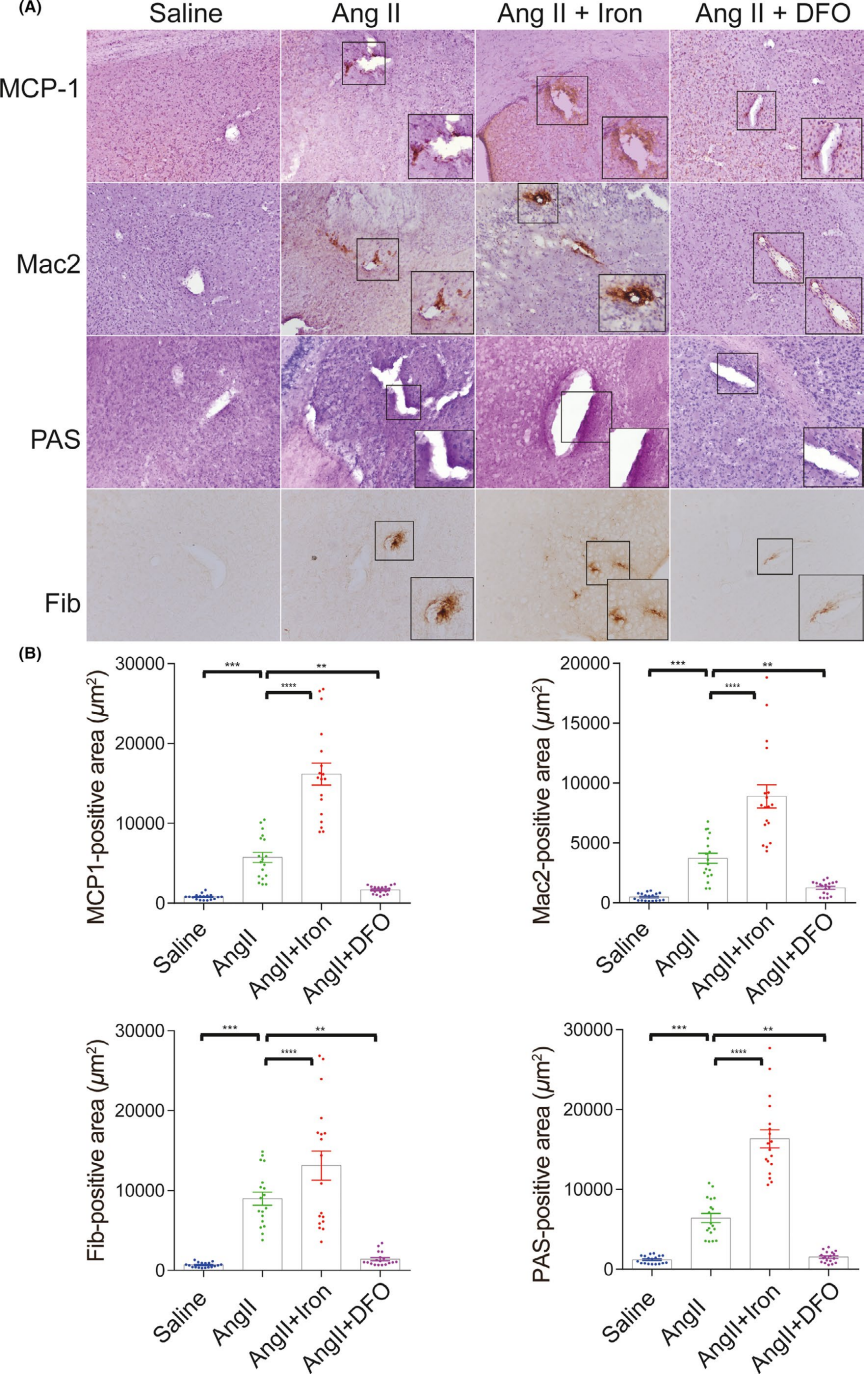
Iron chelation decreased leukocyte recruitment and blood–brain barrier leakage in angiotensin II (Ang II)‐induced hypertensive mice. (A) Representative images and quantification (B) of immunochemistry for the MCP‐1, Mac2, Fib (fibrinogen), and PAS staining at 28 days after saline, Ang II, Ang II +iron dextran or Ang II +DFO treatment. All the images were taken at a magnification of 200 times. Three representative images were quantified for each sample (with a sample size of 6 for each group). The unit of positive signal area is square micron (μm^2^). *p < 0.05, **p < 0.01, ***p < 0.005, ****p < 0.001

Blood–brain barrier (BBB) disruption is a critical pathophysiological change that occurs after ICH and is considered to be closely related to HE.^31^ However, it is still unclear whether pre‐ICH BBB status might influence HE. Ang II infusion resulted in significant BBB leakage, as demonstrated by fibrinogen deposition and plasma extravasation (PAS staining), and promoted MCP‐1and MAC2 accumulation on the vessel wall. Iron overload increased BBB leakage and leukocyte recruitment, while iron chelation preserved BBB integrity and decreased perivascular MCP‐1 and MAC2 accumulation (Figure 5). Together with the secondary bleeding results, these findings suggested that mice with more severe BBB leakage exhibited exacerbated secondary bleeding, implying that pre‐ICH BBB status influenced HE.

### 3.5 Iron chelation during the process of Ang II‐induced hypertension attenuated glial Cell accumulation and MMP9 expression upregulation after ICH modeling

**FIGURE 6:**
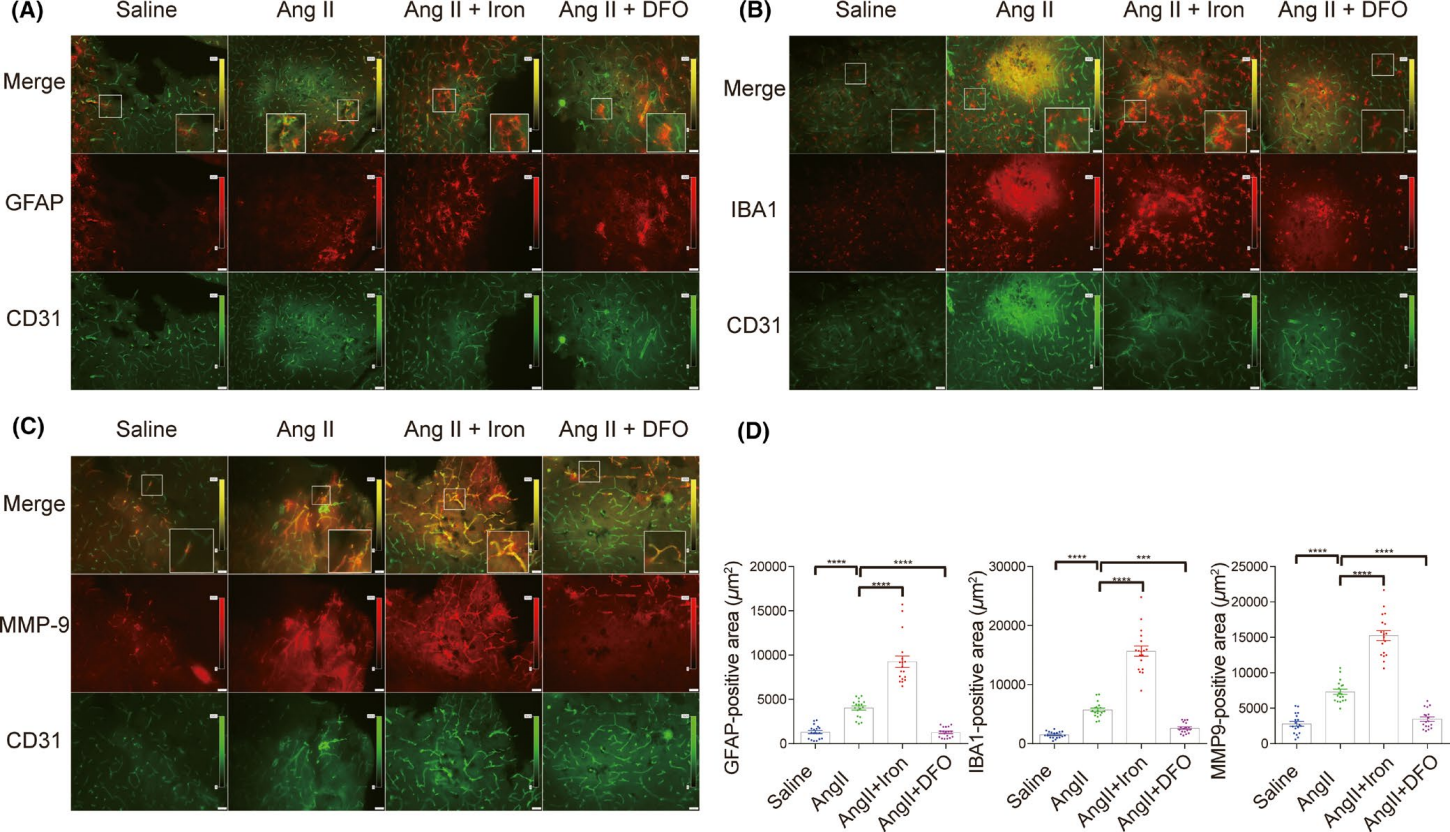
Iron chelation attenuated glial cell accumulation and MMP9 expression upregulation in angiotensin II‐induced hypertensive mice after intracerebral hemorrhage (ICH) modeling. (A–C) Representative images and quantification (D) of immunofluorescence for the glial cell markers (GFAP and IBA‐1) and MMP9 at 24 h after ICH modeling. All the images were taken at a magnification of 200 times. Three representative images were quantified for each sample (with a sample size of 6 for each group). The unit of positive signal area is square micron (μm^2^). *p < 0.05, **p < 0.01, ***p < 0.005, ****p < 0.001

In the acute phase of ICH, resident astrocytes and microglia are involved in inflammatory responses.^32^ Astrocytes accumulate in the perihematomal region (1–3 days after ICH) much earlier than microglia accumulate (3–7 days after ICH).^33^ Nevertheless, the status of glial cells within the first 24 h of ICH in Ang II‐induced hypertensive mice has not been fully studied. Twenty‐four hours after ICH modeling, upregulation of the expression of the glial cell markers GFAP and IBA‐1 and MMP9 was observed around the secondary bleeding sites in the Ang II‐infused mice compared with the saline‐infused mice. Iron overload further enhanced the glial cell accumulation and MMP9 expression upregulation. In contrast, iron chelation significantly decreased glial cell accumulation and MMP9 expression upregulation (Figure 6). We labeled the glial cells with combinations of molecular markers (IBA 1+ CD68 for microglia, GFAP +AQP 4 and GFAP +C3 for astrocytes). As seen in the supplemental Figure 1, the IBA 1+ CD68+ cells were only seen in the Ang II+Iron group (Figure S1 C). Besides, a few GFAP +AQP 4+ cells were only seen in the Ang II+Iron group (Figure S1 A). GFAP+C3+ positive cells were not seen among the four groups (Figure S1 B). So, the status of glial cells at 24 h after ICH in Ang II‐induced hypertensive mice was speculated as glial accumulation rather than glial activation.

## 4 CONCLUSION

In the present study, we confirmed for the first time that the iron content increases in the brain tissues of Ang II‐induced hypertensive mice and that iron intervention significantly affects the extent of secondary bleeding in Ang II‐induced hypertensive mice after ICH modeling. Mechanistically, iron intervention affects the degree of cerebral vascular injury in Ang II‐induced hypertensive mice as well as the accumulation of glial cells and MMP9 expression in Ang II‐induced hypertensive mice after ICH modeling. These findings provide experimental evidence that iron plays a key role in secondary hemorrhage in Ang II‐induced hypertensive mice and that iron might serve as a potential therapeutic target.

Previously generated animal models of secondary bleeding were generated by modifying protocols for constructing ICH models by intrastriatal injection of collagenase/blood. In collagenase‐induced ICH models treated with anticoagulants or subjected to hyperglycemia, collagenase dissolves the vessels, leading to continuous expansion of the hematoma, which might be inappropriate for the simulation of secondary bleeding.^31^, ^34^ Intrastriatal injection of blood into hyperglycemic/hypertensive mice indeed causes significant HE^35^, ^36^; however, it is difficult to distinguish the initial hematoma from the secondary hemorrhage. Intrastriatal injection of a liquid polymer simulates an initial hematoma that is distinguishable from the secondary hemorrhage.^6^ In our study, we further modified the model by inducing ICH in Ang II‐induced hypertensive mice to mimic HE most often seen in hypertensive ICH. Also, our study has limitations. Only the secondary hemorrhage of 24 h after ICH was evaluated, which might not reflect the dynamic evolution of the hematoma. Future work will examine the same animal models longitudinally using MRI methodologies that can help monitor the induction, expansion, and absorption of ICH over a long period of time as previously reported.^37^, ^38^, ^39^

Ang II‐induced hypertension might cause the expansion of the secondary hemorrhage through several mechanisms. It has been reported to increase the hematoma volume by inducing inflammation and apoptosis of cerebral VSMCs via the TNF‐α‐dependent nuclear factor‐kappaB pathway.^40^ It might also enhance secondary bleeding by elevating blood pressure since both preexisting hypertension and acute hypertension in response to ICH can contribute to HE.^41^ Our study confirmed that iron accumulation in the brains and secondary bleeding increased after ICH modeling in Ang II‐induced hypertensive mice. Iron intervention significantly affected the degree of secondary bleeding. Thus, our data confirmed the involvement of iron in secondary bleeding in Ang II‐induced hypertensive mice from an experimental perspective.

Increased iron accumulation in the brains of Ang II‐induced hypertensive mice might contribute to secondary bleeding by decreasing the integrity of the blood vessels. Previous studies identified VSMC apoptosis as a characteristic pathology of vascular rupture in patients with hypertensive cerebral hemorrhage,^42^ indicating the key role of VSMCs in vascular integrity. In Ang II‐induced hypertensive mice, we observed the loss of contractile VSMCs, and iron chelation rescued the loss of contractile VSMCs and decreased secondary bleeding. In addition, BBB dysfunction easily leads to vessel rupture and precedes ICH.^43^ Prevention of BBB leakage by BB‐94 was reported to reduce the hematoma volume, suggesting that BBB disruption is a promoter of HE.^36^ We also observed increased BBB leakage in Ang II‐induced hypertensive mice, and iron chelation decreased BBB leakage and secondary bleeding.

Iron overload during the process of Ang II‐induced hypertension might exacerbates brain injury after ICH by inflammatory activation. Both ICH‐related systemic inflammation^44^, ^45^ and microglia/macrophage‐mediated inflammation activation^46^ play an important role in brain injury, recovery, and stroke outcome. In our study, increased vascular inflammation (indicated by MCP‐1‐and Mac2‐positive cells and IBA‐1 positive cells) were observed in Ang II‐infused mice, while, iron chelation decreased inflammatory activation and secondary bleeding. Effects of iron overload in this animal model on systemic inflammation and its influence on brain injury, recovery, and stroke outcome deserves further investigation.

In addition, after ICH modeling, we observed obvious accumulation of astrocytes, the inhibition of which has been reported to decrease HE and BBB destruction in collagenase‐induced ICH rats.^47^ Moreover, the accumulation of microglia (1 day after ICH) happened earlier than previously reported (3–7 days after ICH).^33^ In our study, iron intervention also influenced the upregulation of MMP9, which might degrade vascular laminin and collagen IV and corrode the vessel wall^48^ and is associated with ICH enlargement and perihematoma edema.^49^

The mechanisms of increased iron accumulation in the brain tissues of Ang II‐induced hypertensive mice remain unclear. Chronic Ang II administration has been reported to upregulate the expression of the duodenal iron transporter ferroportin1, leading to increased iron absorption into the blood and macrophage iron content.^50^ Ang II‐induced iron deposition in the rat liver, heart, and aorta is presumed to be mediated by the upregulation of the expression of heme oxygenase‐1(HO‐1), which participates in the cleavage of the heme ring, producing biliverdin, CO, and ferrous Fe.^10^, ^24^, ^25^ Intraventricular administration of 5 μg of AII induces significant increases in levels of TfR, DMT1, and Fpn1. However, the levels of ferritin do not increase significantly.^51^ Few studies have assessed the effect of peripheral administration of Ang II on brain iron hemostasis. Our study demonstrated for the first time that the iron content increases in the brain tissues of Ang II‐induced hypertensive mice. There are two possible factors that might contribute to the iron deposition observed in the brains of the angiotensin‐infused mice. First, iron might enter the brain through iron transporters since Ang II has been reported to facilitated the protein expression of the transferrin receptor, divalent metal transporter 1, and ferroportin1 in human glomerular endothelial cells.^52^ Second, the increased serum iron might enter the brain through the leaking brain‐blood barrier. Further study is needed to elucidate more details about the mechanism.

In conclusion, iron accumulation in the brain tissues of Ang II‐induced hypertensive mice might exacerbate the loss of contractile VSMCs, enhance perivascular inflammation and BBB leakage, and intensify the glial accumulation and MMP9 expression upregulation after ICH. These factors might lead to decreased vascular integrity and increased vulnerability to rupture, ultimately causing more secondary bleeding and subsequent HE after ICH. Iron chelation might serve as a potential therapeutic strategy.

## CONFLICTS OF INTEREST

The authors declare no conflicts of interest.

## AUTHORS’ CONTRIBUTIONS

J.W. and W.C. designed the experiments. J.W., M.X., X.T., C.L., Y.Y., C.G., H.G., and B.W. preformed the experiments and discussed the results. J.W. and X.T. collected and analyzed all the present data. W.C., and H.F. wrote the draft and worked on the manuscript revision. All authors read and approved the final manuscript.

## Supporting information

Figure S1

Supplementary Material

## Data Availability

The data that support the findings of this study are available from the corresponding author upon reasonable request.
