# Supplementary figures and images for: Iron chelation suppresses secondary bleeding after intracerebral hemorrhage in angiotensin II‐infused mice
Source: CNS Neurosci Ther. 2021 Aug 4;27(11):1327–38. doi: 10.1111/cns.13706 (PMC8504530; doi:10.1111/cns.13706)

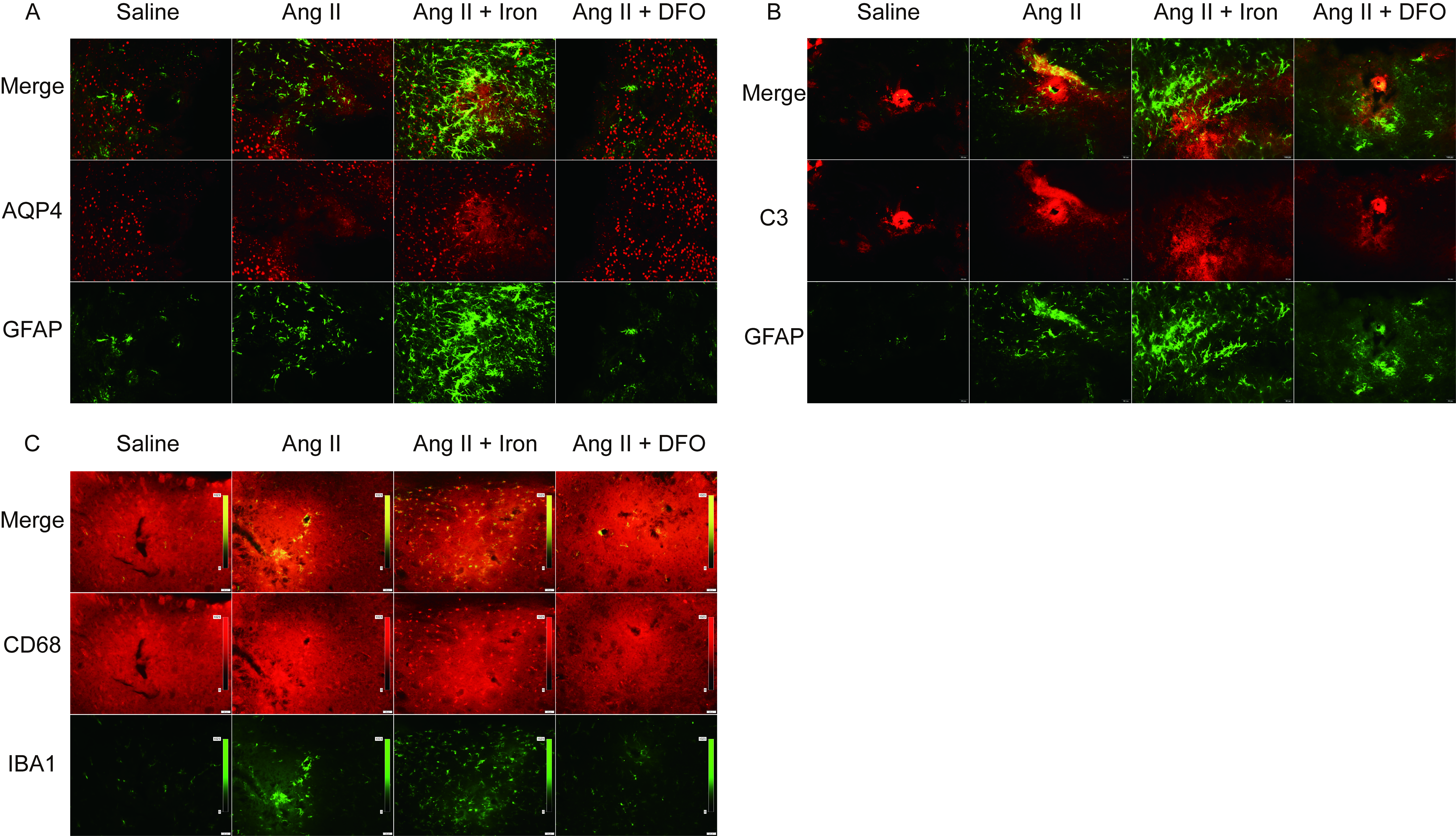

Supplement: Supplementary file 1 — Figure S1 [file CNS-27-1327-s001.tif]
