# Supplementary material for: Iron chelation suppresses secondary bleeding after intracerebral hemorrhage in angiotensin II‐infused mice
Source: CNS Neurosci Ther. 2021 Aug 4;27(11):1327–38. doi: 10.1111/cns.13706 (PMC8504530; doi:10.1111/cns.13706)
Supplement: Supplementary file 2 — Supplementary Material [file CNS-27-1327-s002.pdf]

Full unedited blot for Figure 1 C

GAPDH
36KDa

FTH
21KDa

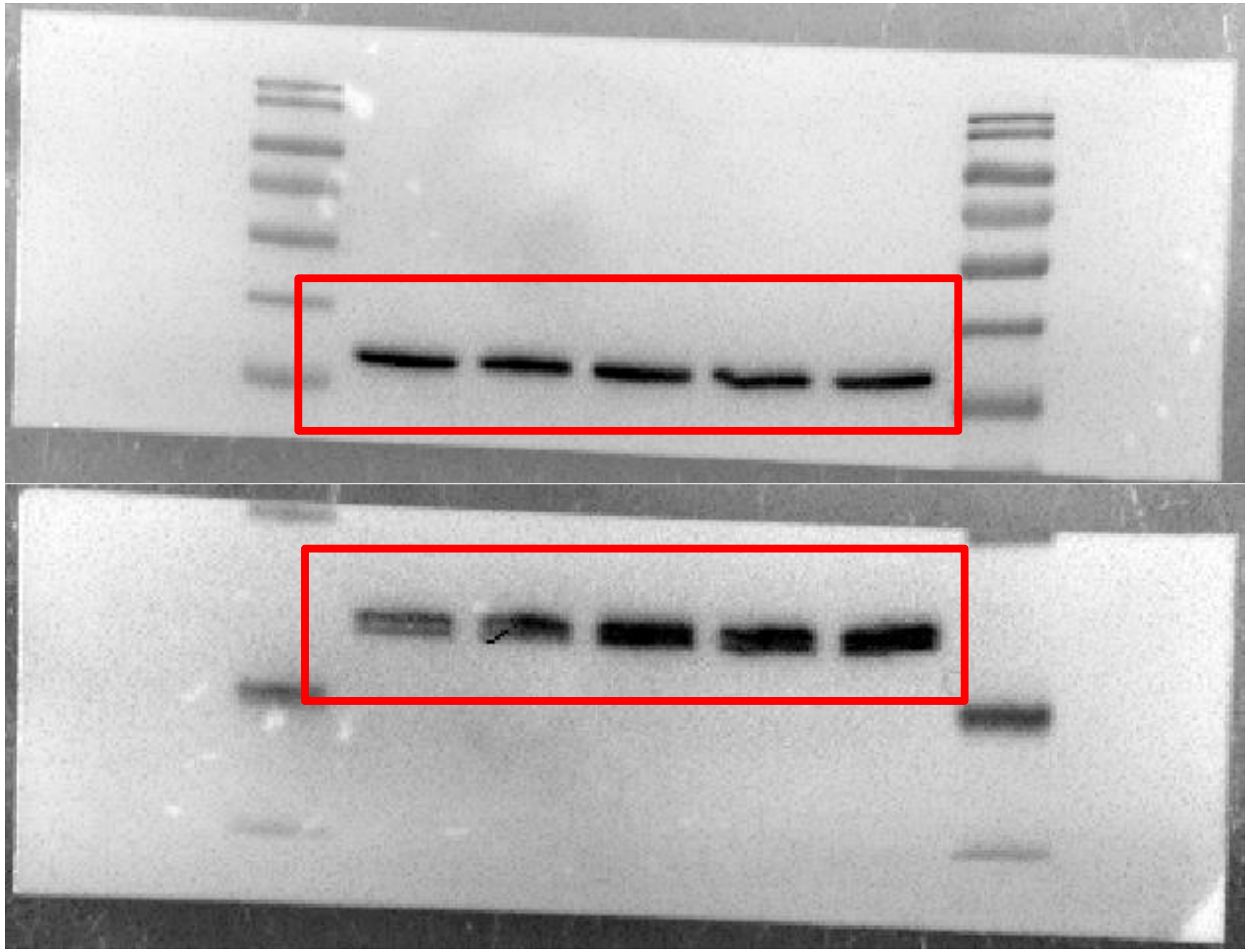

180 KDa
130 KDa
100 KDa
70 KDa
55 KDa
40 KDa
35 KDa
25 KDa
15 KDa
10 KDa

Full unedited blot for Figure 1 D

GAPDH
36KDa

FTL
21KDa

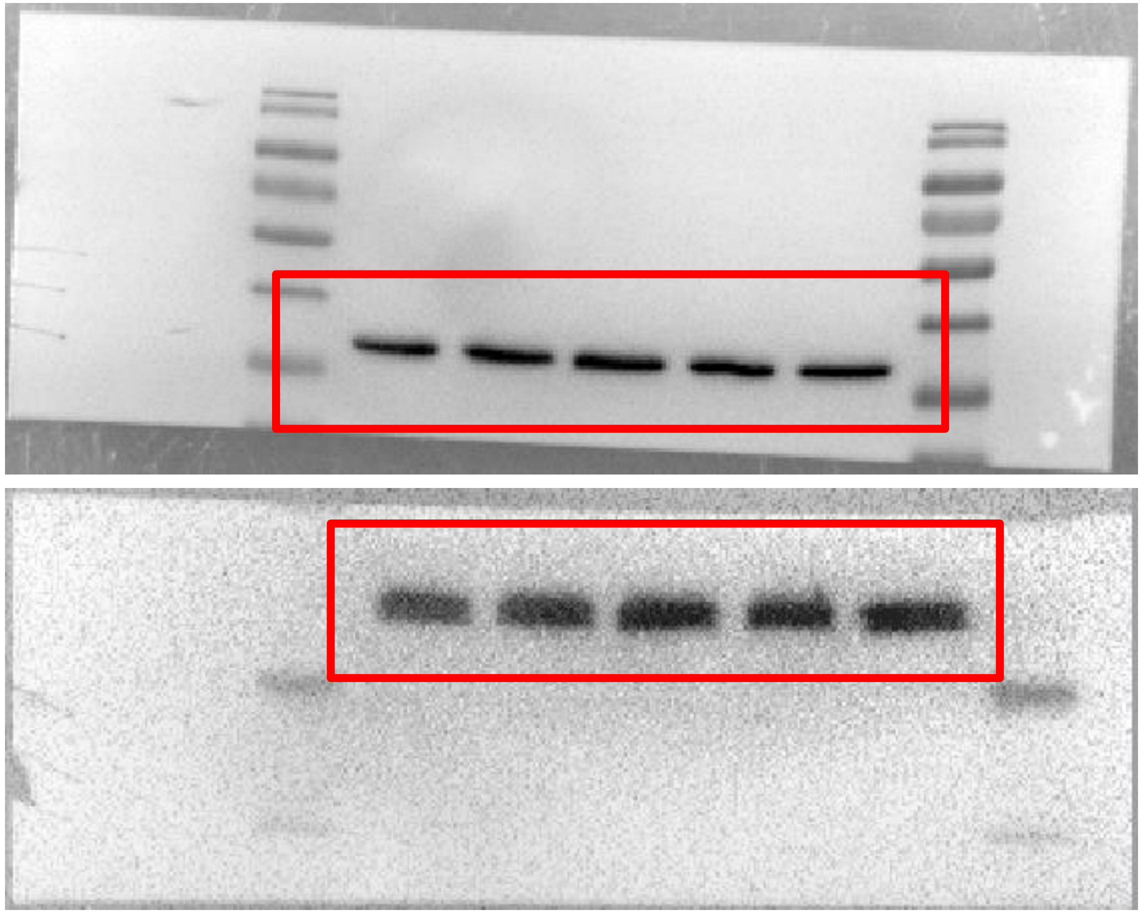

180 KDa
130 KDa
100 KDa
70 KDa
55 KDa
40 KDa
35 KDa
25 KDa
15 KDa
10 KDa

Full unedited blot for Figure 4 A

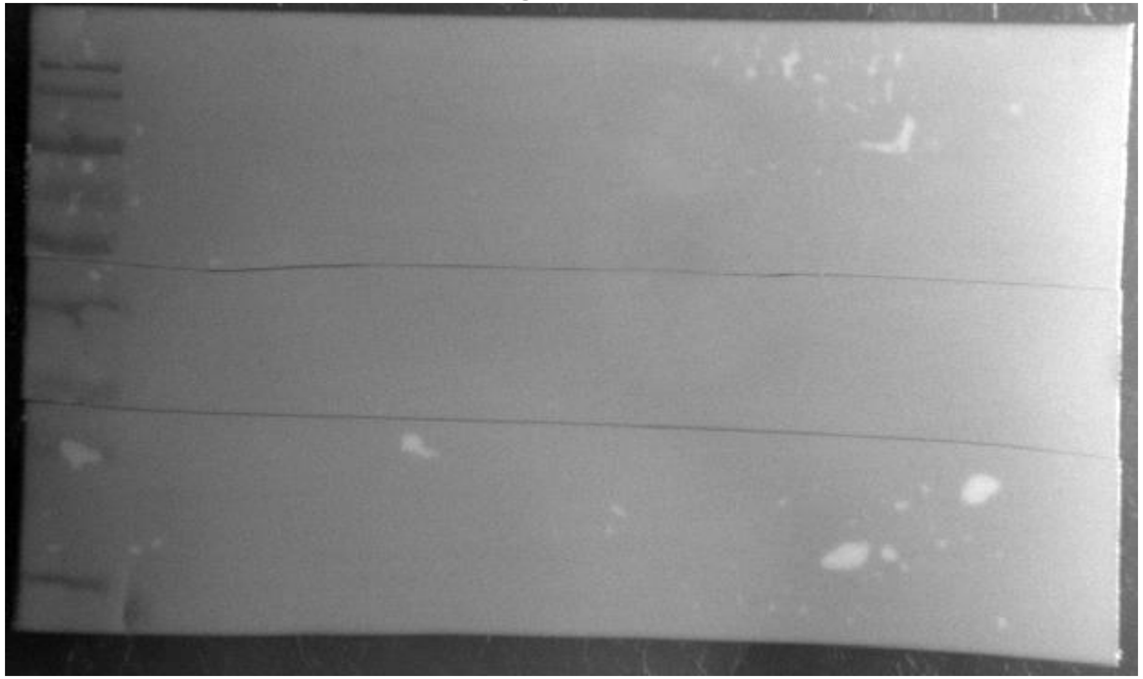

180 KDa
130 KDa
100 KDa
70 KDa
55 KDa
40 KDa
35 KDa
25 KDa
15 KDa

GAPDH
36KDa

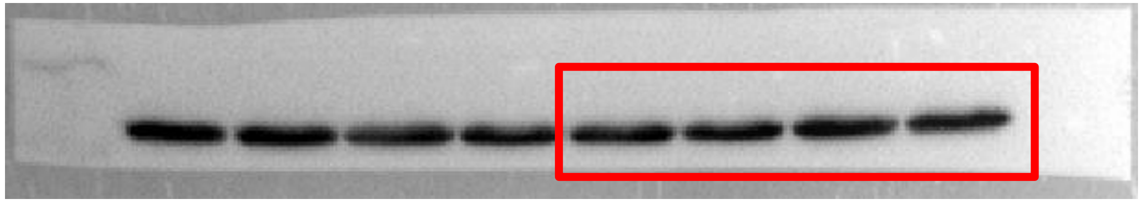

Ferritin
21KDa

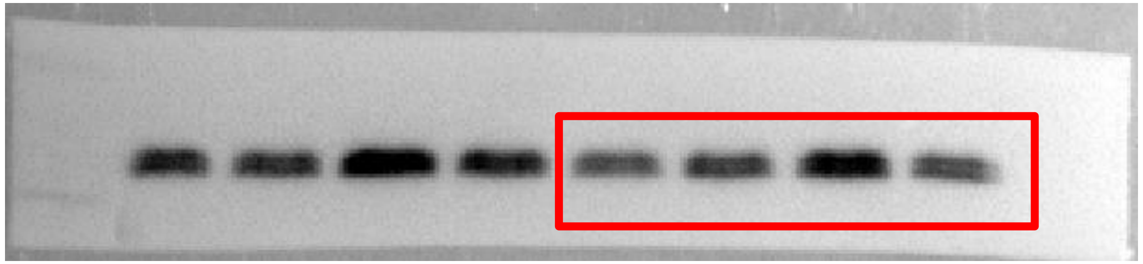

Full unedited blot for Figure 4 C

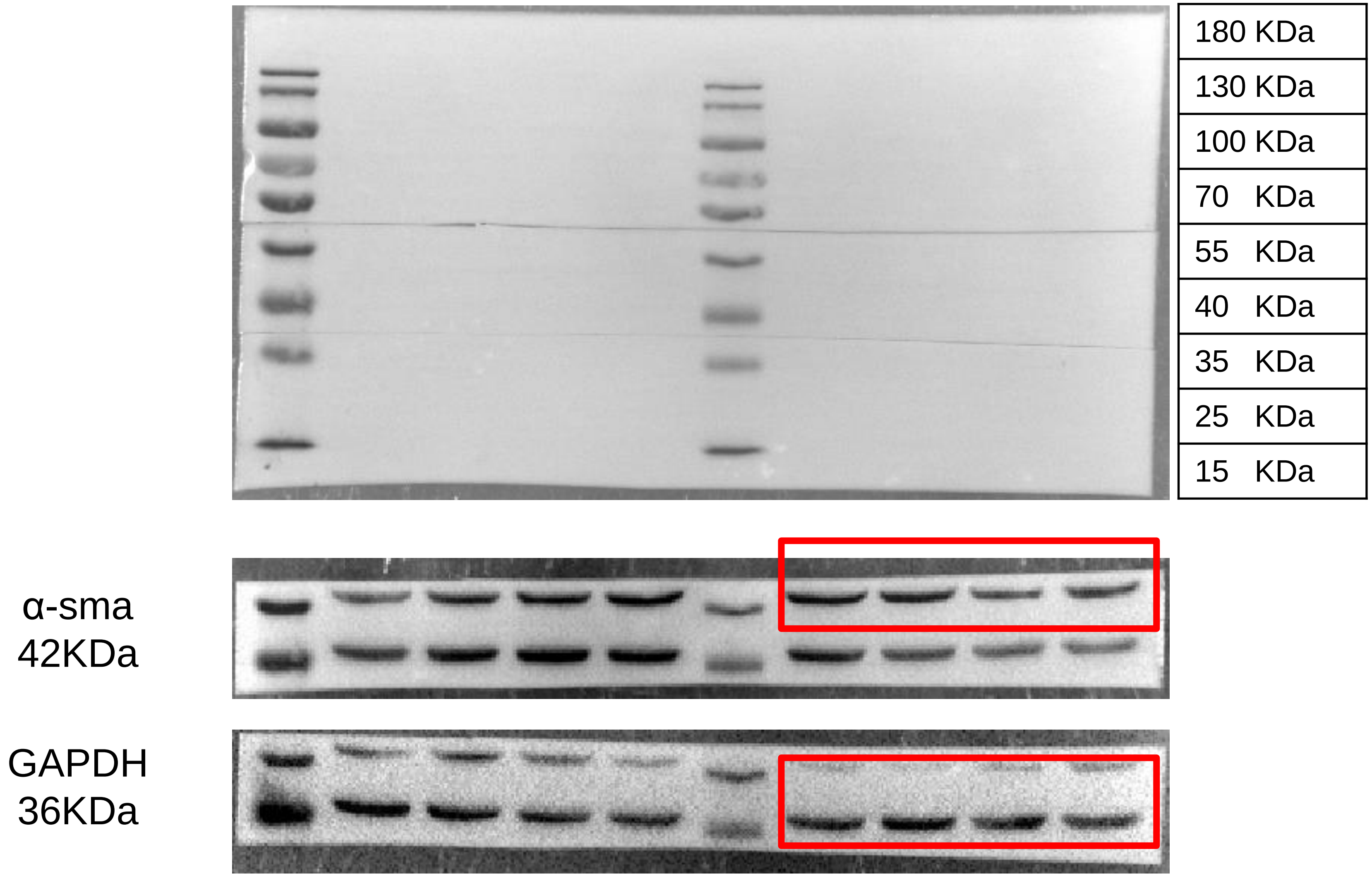

Full unedited blot for Figure 4 E

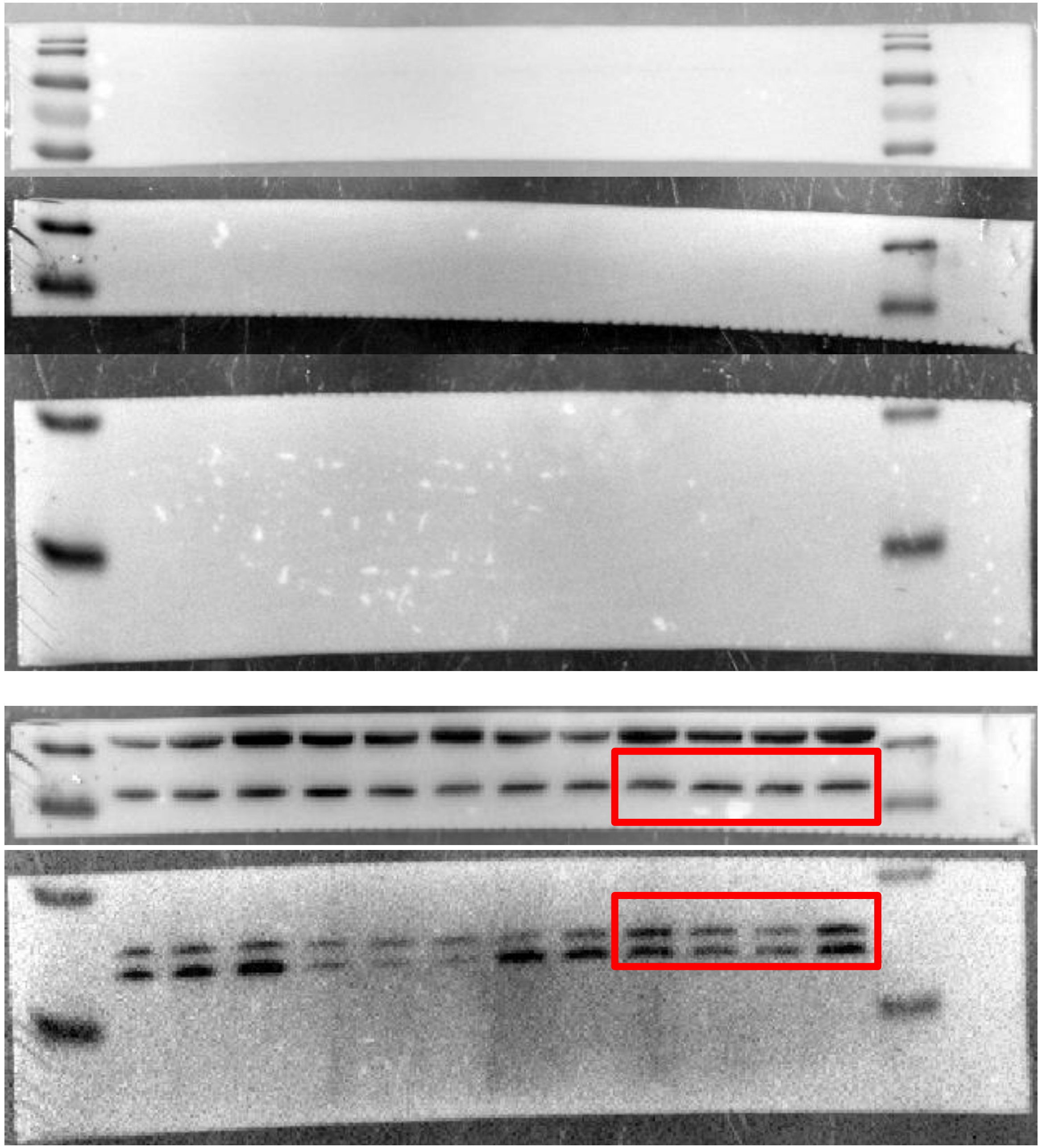

180 KDa
130 KDa
100 KDa
70 KDa
55 KDa
40 KDa
35 KDa
25 KDa
15 KDa

GAPDH
36KDa

Sm22α
22KDa
